# Furin extracellularly cleaves secreted PTENα/β to generate C-terminal fragment with a tumor-suppressive role

**DOI:** 10.1038/s41419-022-04988-2

**Published:** 2022-06-06

**Authors:** Cheng Zhang, Hong-Ming Ma, Shuang-Shu Dong, Na Zhang, Ping He, Meng-Kai Ge, Li Xia, Jian-Xiu Yu, Qiang Xia, Guo-Qiang Chen, Shao-Ming Shen

**Affiliations:** 1 Department of Pathophysiology, Key Laboratory of Cell Differentiation and Apoptosis of Chinese Ministry of Education, Ruijin Hospital, Shanghai Jiao Tong University School of Medicine (SJTU-SM), 200025 Shanghai, China; 2 State Key Laboratory of Cell Biology, Center for Excellence in Molecular Cell Science, Shanghai Institute of Biochemistry and Cell Biology, University of Chinese Academy of Sciences, Chinese Academy of Sciences, 200031 Shanghai, China; 3 State Key Laboratory of Oncogenes and Related Genes, and Chinese Academy of Medical Sciences Research Unit (NO .2019RU043), Renji hospital, SJTU-SM, 200127 Shanghai, China

**Keywords:** Cancer, Oncogenesis

## Abstract

PTENα and PTENβ (PTENα/β), two long translational variants of phosphatase and tensin homolog on chromosome 10 (PTEN), exert distinct roles from canonical PTEN, including promoting carcinogenesis and accelerating immune-resistant cancer progression. However, their roles in carcinogenesis remain greatly unknown. Herein, we report that, after secreting into the extracellular space, PTENα/β proteins are efficiently cleaved into a short N-terminal and a long C-terminal fragment by the proprotein convertase Furin at a polyarginine stretch in their N-terminal extensions. Although secreted PTENα/β and their cleaved fragment cannot enter cells, treatment of the purified C-terminal fragment but not cleavage-resistant mutants of PTENα exerts a tumor-suppressive role in vivo. As a result, overexpression of cleavage-resistant PTENα mutants manifest a tumor-promoting role more profound than that of wild-type PTENα. In line with these, the C-terminal fragment is significantly downregulated in liver cancer tissues compared to paired normal tissues, which is consistent with the downregulated expression of Furin. Collectively, we show that extracellular PTENα/β present opposite effects on carcinogenesis from intracellular PTENα/β, and propose that the tumor-suppressive C-terminal fragment of PTENα/β might be used as exogenous agent to treat cancer.

## Introduction

Phosphatase and tensin homolog on chromosome 10 (PTEN), a tumor suppressor frequently lost or mutated in human sporadic cancers, exerts both phosphatase-dependent and -independent activities in the cell and governs a variety of biological processes [1–5]. Besides canonical PTEN (hereafter called PTEN), several evolutionarily conserved translational variants of PTEN, including PTENα (PTEN-long/PTEN-L), PTENβ, and PTENε, were identified [6–10]. Human PTENα, PTENβ, and PTENε are translated respectively from alternative translation initiation codons CUG^513^, AUU^594^ and CUG^816^ of PTEN mRNA that are 5’ of and in-frame with the canonical translation initiation codon AUG^1032^ of PTEN, thus respectively adding a N-terminal extension (NTE) including 173, 146 and 72-amino acid to the 403 amino acids of PTEN. Their levels are much lower than that of PTEN, consistent with the concept that use of non-AUG start codons is typically less efficient than mRNA translation from canonical AUG translation start sites [11, 12]. The initial report showed that PTEN-L/PTENα is a membrane-permeable lipid phosphatase that is secreted from cells and can enter other cells to functions as a secretory PI3K antagonist and proposed that PTENα might be used as exogenous agent to treat cancer [6]. PTENα was also reported to localize at the outer mitochondrial membrane and contribute to mitophagy as a protein phosphatase for ubiquitin [13] and through promotion of PARK2 recruitment to damaged mitochondria [14]. Also, PTENα induces cytochrome c oxidase activity and ATP production in mitochondria and its somatic deletion impairs mitochondrial respiratory chain function [9]. PTENβ was reported to localize predominantly in the nucleolus, and physically associates with and dephosphorylates nucleolin. Accordingly, disruption of PTENβ alters rDNA transcription and promotes ribosomal biogenesis [7]. However, we reported that both PTENα and PTENβ (PTENα/β) manifest prominent nuclear localization due to the presence of a canonical nuclear localization signal in their NTEs, and exert tumor-promoting roles in liver cancer cells through the direct interaction of their NTEs with the histone H3 lysine 4 (H3K4) presenter WDR5 in the nucleus to promote H3K4 trimethylation and maintain a tumor-promoting signature [15]. Recently, PTENα was also shown to remain active in cancer-bearing stop-gained PTEN mutations, and lead to T cell dysfunction and accelerate immune-resistant cancer progression [16]. Totally, roles of PTENα/β in carcinogenesis are more complicated and remain to be further explored.

The NTEs of PTEN variants subject them to differential regulations and enable them to play distinct roles from that of canonical PTEN [6–9]. A bioinformatic analysis on the structural properties of the NTE of PTENα revealed that it is enriched in post-translational modification sites and protein-binding motifs indicating the probable role of enzymatic modifications and protein-protein interactions in the function of PTENα [17]. Indeed, the binding of ubiquitin E3 ligase FBXW11 or deubiquitinase USP9X to the NTEs of PTENα/β regulates their stability without influences on PTEN [15]. We reasoned that experimental characterization of these regulatory and functional motifs in NTEs of PTENα/β will serve as an effectual way to further understand PTENα/β.

Herein, we report that the secreted PTENα/β proteins are efficiently cleaved by the proprotein convertase Furin in the extracellular space, and the cleaved C-terminal fragment but not full-length PTENα exerts a tumor-suppressive role. These findings dissect the contribution of extracellular and intracellular PTENα to its overall tumor-promoting role, and propose that the tumor-suppressive C-terminal fragment might be used as exogenous agent to treat cancer.

## Results

### PTENα/β are cleaved within their NTEs in vivo

**Fig. 1:**
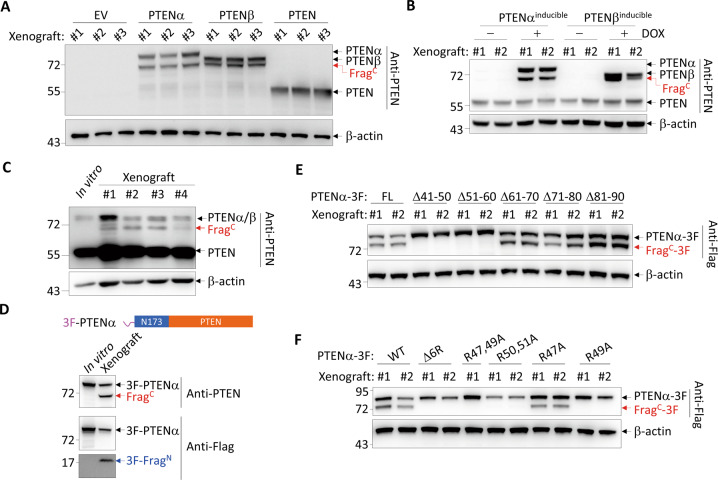
PTENα/β are cleaved within their NTEs. **A**, **B** Xenografts derived from *PTEN*-knockout SMMC-7721 cells stably expressing EV, PTENα, PTENβ, or PTEN (**A**) and from SMMC-7721 cells with inducible expression of PTENα or PTENβ under the control of a doxycycline-inducible expression system (**B**) were subjected to Western blot for proteins as indicated. (**C**) Western blot analysis in the lysates of in vitro-cultured SMMC-7721 cell line and its 4 xenografts. **D** Schematics of N-terminal 3×Flag-tagged PTENα (3F-PTENα) (top). Western blot analysis for indicated proteins in the lysates of in vitro-cultured *PTEN*-knockout SMMC-7721 cell line stably expressing 3F-PTENα and the xenograft derived from it (bottom). **E**, **F** Western blot analysis for indicated proteins in xenografts derived from *PTEN*-knockout SMMC-7721 cells stably expressing indicated PTENα derivatives. EV, empty vector; 3 F, 3×Flag.

We previously reported the tumor-promoting roles of PTENα/β in liver cancer [15]. Here, in the xenografts derived from *PTEN*-knockout hepatocellular carcinoma SMMC-7721 cells (SMMC-7721ΔPTEN) with re-expression of PTEN, PTENα, or PTENβ, we unexpectedly detected an additional band at ~ 68 kD under the expression of PTENα or PTENβ but not PTEN by Western blot analysis with an antibody against the C-terminus of PTEN (Fig. 1A). This band could also be seen in xenografts derived from SMMC-7721 cells with inducible expression of PTENα and PTENβ under the control of a doxycycline-inducible expression system (Fig. 1B), and in xenografts derived from human colon cancer SW620, human lung cancer NCI-H441, human liver cancer Huh7 and human pancreatic cancer MiaPaCa2 with stable exogenous PTENα expression (Fig. S1A). In addition, this band was observed in xenografts derived from parental SMMC-7721 cells expressing only endogenous PTENα/β (Fig. 1C). Because the molecular weights of PTENβ and this band are too close and it is especially hard to tell them apart at their low abundant endogenous levels, we used the previously described electrophoresis strategy [15] that does not separate PTENα and PTENβ to detect these proteins at endogenous level here and hereafter. In contrast, the band could not be detected in cell lysates of in vitro-cultured cell lines from which these xenografts were derived (Fig. 1C and S1B).

To understand whether this band belongs to PTEN proteoform, we used PTENα as a representative, and generated *PTEN*-knockout SMMC-7721 cells expressing N-terminal 3×Flag (3 F)-tagged PTENα. Unexpectedly, Western blot analysis of the xenograft derived from this cell line showed that anti-PTEN antibody rather than anti-Flag antibody detected this band, while anti-Flag antibody detected another band at ~19 kD (Fig. 1D). Intriguingly, these two additional bands could not be detected in cell lysates of in vitro-cultured cells (Fig. 1D). These results indicate that PTENα was cleaved in xenografts to produce a short N-terminal and a long C-terminal fragment, which we designated respectively as Frag^N^ and Frag^C^.

Considering that the molecular weight of Frag^C^ was ~2 kD smaller than that of PTENβ (Fig. 1A, B), we predicted the cleavage site to be within the range of 10 to 50 residues after the translation initiation site of PTENβ, roughly corresponding to residues 41 to 90 in PTENα (Fig. S1C). Therefore, a series of deletions were made to this region based on PTENα protein sequence. Western blot analysis with xenografts expressing these mutants showed that deletion of either residues 41-50 or 51-60 abolished PTENα cleavage (Fig. 1E). Because these two regions contain a polyarginine stretch (residues 47 to 52) (Fig. S1C), we deleted this polyarginine stretch (PTENα-Δ6 R), and found the deleted mutant of PTENα almost completely abolished PTENα cleavage (Fig. 1F). Moreover, single mutation of arginine 49 (PTENα-R49A) or double mutations of arginines 50 and 51 (PTENα-R50, 51 A) was enough to phenocopy the deletion of the whole stretch (Fig. 1F). Thus, our results demonstrate that PTENα is cleaved at a polyarginine stretch within its NTE.

### Cleavage of PTENα occurs extracellularly

**Fig. 2:**
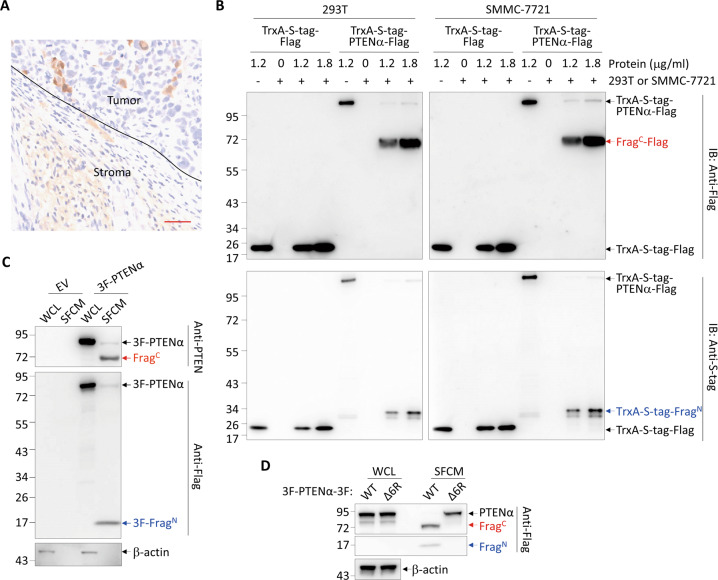
PTENα is efficiently cleaved in the extracellular space. **A** Representative image of the immunohistochemistry staining with an anti-Flag antibody in the section of a xenograft derived from SMMC-7721ΔPTEN cells expressing PTENα-3F. Scale bar, 50 μm. **B** Bacterially purified TrxA-S-tag-Flag or TrxA-S-tag-PTENα-Flag was co-cultured with and without 293 T or SMMC-7721 cells for 4 hours in SFCM. Western blot analysis for the indicated proteins in the SFCM was performed. **C**, **D** Western blot analysis for the indicated proteins in the WCL and SFCM of 293 T cells transfected with EV or 3F-PTENα (**C**) and PTENα-WT or PTENαΔ6 R tagged by 3×Flag at both ends (**D**). 3 F 3×Flag, WCL whole-cell lysate, SFCM serum-free conditioned medium.

Immunohistochemistry staining of xenografts derived from *PTEN*-knockout SMMC-7721 cells expressing PTENα-3F with an anti-Flag antibody not only detected positive staining in the tumor tissues, but also in the mouse-derived stroma, indicating that PTENα-3F is secreted by tumor cells into the extracellular space (Fig. 2A). Considering PTENα/β fragments were only detected in xenografts but not in in vitro-cultured cell lysates, we reasoned that the cleavage might specifically take place in the extracellular space. To test this, bacterially purified TrxA-S-tag-PTENα-Flag together with TrxA-S-tag-Flag as a control were co-cultured with and without 293 T or SMMC-7721 cells for 4 h in the serum-free medium. The results demonstrated that in the presence of 293 T or SMMC-7721, TrxA-S-tag-PTENα-Flag but not TrxA-S-tag-Flag was significantly cleaved into two fragments respectively detected by antibodies against N-terminal S-tag and C-terminal Flag tag in the supernatant (Fig. 2B), indicating that the cleavage of PTENα could efficiently take place in the extracellular space. To test whether this was also true with cell-intrinsic PTENα, Flag-tagged PTENα were transfected into 293 T cells, and whole-cell lysates (WCL), as well as serum-free culture medium (SFCM) were collected and analyzed. The results showed that Frag^N^ and Frag^C^ together with a relatively small amount of full-length PTENα were detected in the SFCM, while only full-length PTENα was seen in WCL (Fig. 2C), indicating that extracellular but not intracellular PTENα was efficiently cleaved. In concert with the finding that PTENα-Δ6 R failed to generate Frag^C^ in xenografts (Fig. 1F), PTENα-Δ6 R was not cleaved in either cell lysate or medium, although it was secreted (Fig. 2D). Taken together, our results indicate that PTENα is cleaved in the extracellular space with high efficiency.

### PTENα is cleaved by Furin

**Fig. 3:**
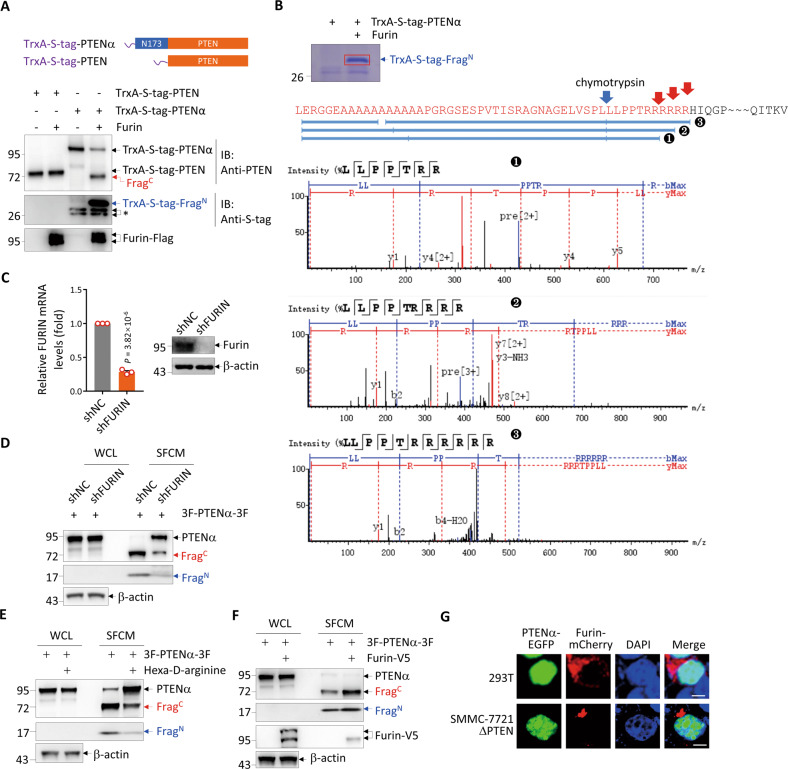
PTENα is cleaved by Furin. **A** Bacterially purified TrxA-S-tag-PTEN or TrxA-S-tag-PTENα (top) were incubated with Flag-tagged Furin purified from 293 T cells, followed by Western blot analysis for the indicated proteins (bottom). Asterisk points to two unknown bands. **B** PTENα was incubated with Furin as in A and the protein products were separated by electrophoresis and stained with Coomassie brilliant blue (top). The Frag^N^ band was cut from the gel and subjected to mass spectrometry analysis after digestion by chymotrypsin. Red arrows indicate the Furin-cleavage sites predicted from three peptides with their tandem mass spectrums shown (bottom). **C** qRT-PCR analysis of *FURIN* mRNA (left) and Western blot analysis for Furin protein (right) in *PTEN*-knockout SMMC-7721 cells with and without knockdown of *FURIN* by shRNA. Data are presented as the means ± SEM (*n* = 3 independent experiments; two-tailed unpaired *t*-test with *P* value shown). **D** Western blot analysis for the indicated proteins in the WCL and SFCM of 3F-PTENα-3F-expressing *PTEN*-knockout SMMC-7721 cells with and without knockdown of *FURIN*. **E**, **F** Western blot analysis for the indicated proteins in the WCL and SFCM of 3F-PTENα-3F-expressing *PTEN*-knockout SMMC-7721 cells with and without treatment by 1 μM Hexa-D-arginine (**E**), and overexpression of V5-tagged Furin (**F**). **G** Representative images of EGFP-tagged PTENα and mCherry-tagged Furin co-transfected in 293 T or *PTEN*-knockout SMMC-7721 cells with re-staining of DAPI. Scale bar represents 10 μm. 3 F 3×Flag, WCL whole-cell lysate, SFCM serum-free conditioned medium, NC negative control.

Bioinformatic prediction proposed that the proprotein convertase Furin is the potential enzyme targeting the polyarginine stretch of PTENα [17]. Interestingly, Furin also exists in the extracellular space [18]. Therefore, we hypothesized that Furin played a role on PTENα/β cleavage. To confirm this, Flag-tagged Furin was expressed and purified from 293 T cells and incubated with bacterially purified PTENα or PTEN. The results showed that incubation of Furin with PTENα but not PTEN generated Frag^C^ and Frag^N^, which were respectively detected by anti-PTEN antibody and an antibody recognizing the S-tag fused to the N-terminus of PTENα (Fig. 3A). Mass spectrometry analysis of the Frag^N^ confirmed that it matched with the N-terminus of PTENα which ended within the polyarginine stretch (Fig. 3B).

Next, we knocked down *FURIN* in 3F-PTENα-3F-expressing *PTEN*-knockout SMMC-7721 cells. The knockdown efficiency was verified by both quantitative real-time PCR and Western blot analysis (Fig. 3C). As expected, *FURIN* knockdown greatly reduced the cleavage of extracellular PTENα (Fig. 3D). In line, treatment with Furin inhibitor hexa-D-arginine [19] showed the similar effect (Fig. 3E). On the contrary, exogenous transfection of Furin, which increased intracellular Furin and its level in the extracellular space, further enhanced the cleavage of extracellular PTENα (Fig. 3F). These results confirmed the involvement of Furin in PTENα cleavage at the cellular level. Collectively, we identified Furin to be responsible for PTENα cleavage in the extracellular space.

To investigate why intracellular PTENα is not cleaved by Furin, we examined the intracellular localizations of PTENα and Furin. As shown in Fig. 3G, while PTENα shows predominant nuclear localization as we previously reported [15], Furin manifests a condensed localization in the cytoplasm, which conforms its localization in the Golgi apparatus as previously reported [20]. The lacking of intracellular co-localization between PTENα and Furin probably explains why intracellular PTENα/β cannot be cleaved by Furin.

### PTENα/β are secreted through two elements in their NTEs

**Fig. 4:**
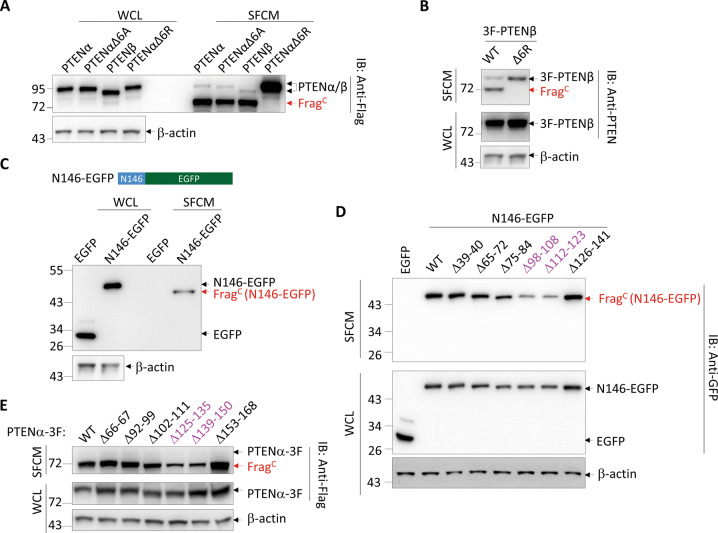
PTENα/β are secreted through two elements in their NTEs. **A**, **B** Western blot analysis for the indicated proteins in the WCL and SFCM of 293 T cells expressing indicated 3F-PTENα-3F derivatives or 3F-PTENβ-3F (**A**) and indicated PTENβ derivatives (**B**). **C** Schematics of N146-EGFP fusion protein (top). Western blot analysis for the indicated proteins with an anti-GFP antibody in 293 T cells expressing EGFP or N146-EGFP (bottom). **D** Western blot analysis for the indicated proteins in 293 T cells expressing EGFP or N146-EGFP derivatives. **E** Western blot analysis for the indicated proteins in the WCL and SFCM of 293 T cells expressing indicated PTENα derivatives. WCL whole-cell lysate, SFCM serum-free conditioned medium, 3 F 3×Flag.

The initial report used SignalP to predict that residues 1-22 in the NTE of PTENα (N173) is a secretion signal peptide [6]. Based on this, they generated the PTENαΔ6A mutant with deletion of 6 alanines in the predicted signal peptide to disrupt PTENα secretion [6] (Fig. S1C). However, here we show that PTENαΔ6A could still be efficiently secreted and extracellularly cleaved (Fig. 4A). On the other hand, PTENβ does not contain this putative secretion signal peptide, but it was still secreted and cleaved at the polyarginine stretch with an efficiency similar to that of PTENα (Fig. 4A, B). Moreover, when the NTE of PTENβ (N146) was fused to EGFP, the resultant N146-EGFP but not EGFP alone was secreted and cleaved (Fig. 4C), indicating the existence of secretion signal sequence(s) in N146. To locate these secretion signal sequence(s), we used PSIPRED to predict the secondary structure of N146, which revealed that the residues 39–40, 65–72, 75–84, 98–108, 112–123, and 126–141 might respectively be folded and form α-helixes. We then established a series of constructs with deletion of these residues on the basis of N146-EGFP (Fig. S2A), and found that the deletion of either residues 98–108 or 112–123 greatly reduced the secretion of N146-EGFP (Fig. 4D). Likewise, deletion of sequences in PTENα corresponding to these two fragments, residues 125–135 and 139–150, also significantly inhibited PTENα secretion (Fig. 4E). Thus, we identified two elements in PTENα/β NTEs to be crucial for their secretion. Of note, these two elements were not predicted to be signal peptide by SignalP (Fig. S2B), raising the possibility that they might not guide secretion through conventional protein secretion pathway.

### The C-terminal fragment of PTENα/β exerts a tumor-suppressive role in vivo

**Fig. 5:**
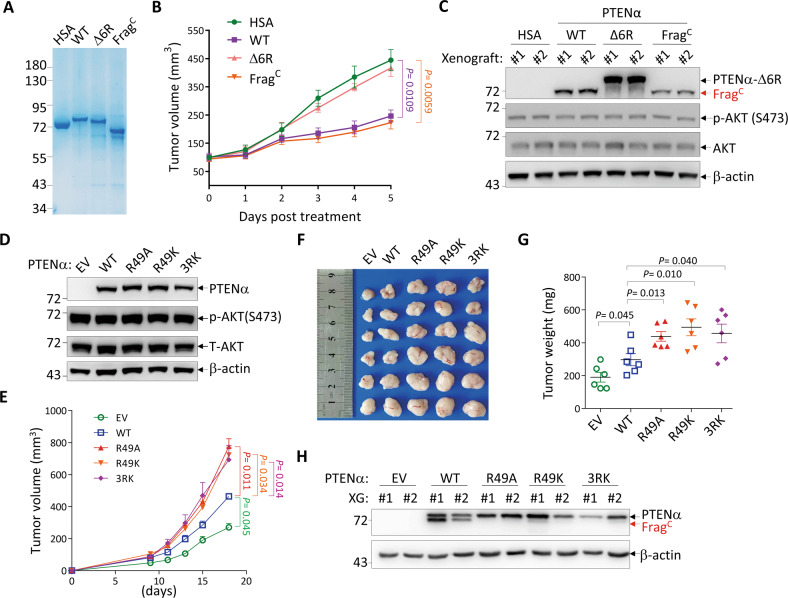
The C-terminal fragment of PTENα exerts a tumor-suppressive role. **A** HSA and bacterially purified PTENα derivatives were visualized by Coomassie blue staining. **B**, **C** *PTEN*-knockout SMMC-7721 cells were subcutaneously injected into nude mice (1.5 × 10^6^ cells per mouse). After tumors reached approximately 100 mm^3^ in volume, HSA and PTENα derivatives were intratumorally injected (10 μg/day for 6 days) and tumor volumes were monitored (**B**). Two tumors from each group were randomly selected for Western blot analysis for the indicated proteins (**C**). Data in B are presented as the mean ± SEM, *n* = 5 biologically independent samples. Statistical significance was determined by two-way ANOVA with *P* values shown. **D**–**H** *PTEN*-knockout SMMC-7721 cells expressing EV or PTENα derivatives (**D**) were subcutaneously injected into nude mice (1.5 × 10^6^ cells per mouse) and tumor volumes were measured at different days (E). On day 18 after subcutaneous injection, tumors were harvested, photographed (**F**) and weighted (**G**), and two tumors from each group were randomly selected for Western blot analysis for the indicated proteins (**H**). Data in E and G are presented as the mean ± SEM, *n* = 6 biologically independent samples. Statistical significance was determined by two-way ANOVA (**E**) and two-tailed unpaired *t*-test (**G**) with *P* values shown. HSA Human Serum Albumin, EV empty vector.

To learn whether and how cleavage affected the behavior of PTENα/β, we focused on Frag^C^, which is longer than Frag^N^ and is generated by both PTENα/β proteins. To address whether Frag^C^ was capable of entering the cell, Frag^C^ (PTENα aa 53-576) as well as wild-type PTENα (PTENα-WT) and PTENα-Δ6 R were bacterially purified (Fig. 5A), and labeled by amine-reactive fluorescent Alexa Fluor 647 dye. After treating SMMC-7721ΔPTEN cells with these proteins for 20 h, no sign of cell entry for all three proteins was observed (Fig. S3A). In contrast, when they were delivered with the help of PULSin, a commercial and highly effective protein delivery reagent in living cells [21], fluorescent proteins could be observed in the cells (Fig. S3A). Western blot analysis of corresponding cell lysates confirmed failure of PTENα derivatives in cell entry without delivery by PULSin (Fig. S3B). We also used the culture medium of 293 T cells expressing EGFP-tagged PTENα-WT or PTENα-Δ6 R to treat SMMC-7721ΔPTEN cells. While both proteins were efficiently expressed in 293 T cells, EGFP-tagged Frag^C^ and PTENα-Δ6 R were respectively detected in the culture mediums (Fig. S3C, D). However, after treating SMMC-7721ΔPTEN cells with these mediums, no trace of EGFP-tagged proteins was detected in SMMC-7721ΔPTEN cells (Fig. S3D, E). Thus, our results indicate that both full-length PTENα and its cleaved C-terminal fragment cannot enter cells.

We then investigated how extracellular Frag^C^ impacted tumorigenesis and whether it behaved differentially to uncleaved PTENα. For this purpose, purified PTENα-WT, PTENα-Δ6 R, and Frag^C^ were injected into xenografts derived from SMMC-7721ΔPTEN cells, with human serum albumin (HSA) as a control. The results showed that treatment with Frag^C^ potently inhibited tumor growth (Fig. 5B). In accordance, PTENα-WT, which was efficiently processed into Frag^C^ in the tumors (Fig. 5C), also inhibited tumor growth to the similar degree as Frag^C^ (Fig. 5B). In contrast, PTENα-Δ6 R, which failed to generate Frag^C^ (Fig. 5C), showed no effect on tumor growth (Fig. 5B). All these results indicate that Frag^C^ rather than full-length PTENα plays a tumor-suppressive role in the extracellular space. In addition, treatment by all three forms of PTENα did not affect the phosphorylation level of tumoral AKT (Fig. 5C), which is in concert with their inability to enter the cell.

### Cleavage-resistant PTENα mutants present enhanced tumor-promoting role

Previously, we reported that overexpression of PTENα/β in SMMC7721ΔPTEN cells promotes tumorigenesis [15]. Hence, we wondered what role extracellular PTENα played in this process. For this purpose, SMMC7721ΔPTEN cells were ectopically expressed with PTENα-WT or three cleavage-resistant PTENα mutants, PTENα-R49A, PTENα-R49K, and PTENα-3RK (with R→K mutation of residues 49, 50, 51 together) (Fig. 5D). These cleavage-resistant mutants of PTENα showed similar nuclear localization (Fig. S3F) and interaction with WDR5 to PTENα-WT (Fig. S3G). Then, these cells were subcutaneously inoculated into nude mice. In accordance with our previous report [15], compared to empty vector, overexpression of PTENα-WT enhanced tumorigenesis, as evidenced by increases in tumor volume and tumor weight (Fig. 5E–G). Intriguingly, all cleavage-resistant mutants of PTENα, which manifested no cleavage during tumor growth (Fig. 5H), showed stronger tumor-promoting effects than that of wild-type PTENα (Fig. 5E–G). These findings suggest that PTENα mutants failing to generate Frag^C^ lose their tumor-suppressive roles in the extracellular space, leading to the manifestation of stronger intracellular tumor-promoting roles.

### Cleavage of PTENα/β is inhibited in liver cancer tissues

**Fig. 6:**
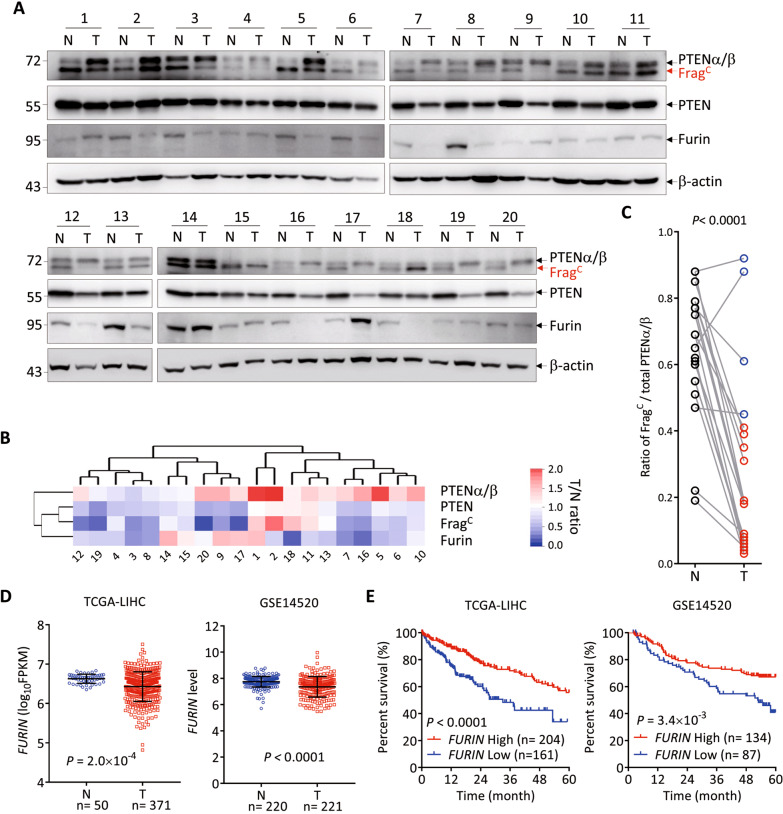
Cleavage of PTENα/β is inhibited in liver cancer tissues. **A**–**C** 20 liver cancer samples with paired tumor and normal tissues were subjected to Western blot analysis for the indicated proteins (**A**). The intensities of the blot bands were quantified by Image J, normalized to that of corresponding β-actin, and the T/N ratio of each protein for each patient was calculated and shown as a clustered heatmap (**B**). The efficiency of PTENα/β cleavage was calculated as the ratio of Frag^C^ versus total PTENα/β (Frag^C^ + full-length PTENα/β) for each sample and compared between the tumor and normal tissue for each patient (**C**). Statistical significance was determined by two-tailed paired *t*-test with *P* values shown. **D** Scatterplot analysis of *FURIN* expression between tumor tissues and non-tumor tissues in TCGA LIHC and GEO GSE14520 data sets. Statistical significance was determined by Mann-Whitney U test (two-sided) with *P* values shown. **E** Kaplan-Meier plot of overall 5-year survival of patients with high or low expression of *FURIN* in the tumor tissues of TCGA LIHC and GEO GSE14520 data sets. Statistical significance was determined by Log-rank test (two-sided).

We then examined the cleavage of PTENα and PTENβ in liver cancer samples. For this purpose, 20 liver cancer samples with paired tumor and normal tissues (Supplemental Spreadsheet 1) were subjected to Western blot analysis. As mentioned above, for detection of endogenous PTENα, PTENβ and Frag^C^, an optimized electrophoresis strategy was employed, which failed to separate PTENα and PTENβ but well separated Frag^C^ from them. The band of unseparated PTENα and PTENβ was designated as PTENα/β, and we found that the band corresponding to Frag^C^ was actually the unknown band we detected in liver cancer samples in our previous study [15]. Compared to normal tissues, PTEN tended to be downregulated in tumor tissues, whereas PTENα/β tended to be upregulated (Fig. 6A, B), a phenomenon that had been described in our previous report [15]. Interestingly, the level of Frag^C^ showed a downregulated trend in tumor tissues compared to paired normal tissues (Fig. 6A, B). We then calculated the percentage of Frag^C^ to total PTENα/β (the total amount of Frag^C^ and full-length PTENα/β) to represent the efficiency of PTENα/β cleavage in each sample, and showed that the efficiency of PTENα/β cleavage was reduced (fold change >1.5) in 16 out of 20 tumor tissues compared to paired normal tissues (Fig. 6C). Consistent with the reduced level of Frag^C^ or efficiency of PTENα/β cleavage in tumor tissues, the expression of Furin was also downregulated (Fig. 6A, B). Downregulated expression of *FURIN* mRNA in tumor tissues was also observed in liver cancer data sets (Fig. 6D), and low *FURIN* expression predicted poorer overall survival of patients with liver cancer (Fig. 6E).

## Discussion

By the identification of an unknown form of PTENα and PTENβ observed in cell line-derived xenograft, here we show that, after secretion, PTENα/β are extracellularly cleaved at a polyarginine stretch within their NTEs by the Furin to generate two fragments, Frag^N^ and Frag^C^. A previous systematic analysis of the PTEN 5′ leader indicates the existence of multiple N-terminally extended PTEN proteoforms, including PTEN-N besides PTEN-L (PTENα), -M (PTENβ) and -O (PTENε) [10]. PTEN-N appears to have a similar molecular weight as Frag^C^. However, their amino acid sequences initiate from different positions. While PTEN-N initiates at Leu43 (based on the protein sequence of PTENα) [10], Frag^C^ begins after Arg47 within the polyarginine stretch cleaved by Furin. Although the secretion of PTENα has been previously reported [6], we failed to validate the previously proposed secretion signal sequence of PTENα [6]. Especially, this sequence is not included in the amino acid sequence of PTENβ, which was similarly secreted and cleaved in the extracellular space as PTENα. Instead, we identified two elements that are present in both PTENα and PTENβ and are crucial for their efficient secretion. Once secreted, due to the high efficiency of Furin-mediated cleavage, both PTENα and PTENβ mainly existed extracellularly as fragments, which was not observed in the previous study [6]. By directly treating tumors with different forms of PTENα, our results showed that the C-terminal fragment instead of the uncleaved full-length PTENα exerts a tumor-suppressive role, emphasizing the critical role of Furin-mediated cleavage for PTENα/β to be functional. Finally, unlike the previous report showing that secreted PTENα was taken up directly by other cells to antagonize PI3K signaling and induced tumor cell death [6], our results failed to show cell entry of either full-length PTENα or the C-terminal fragment, or their impact on phosphorylation level of AKT. This is also compatible with our finding that the polyarginine stretch of PTENα is efficiently cleaved and disrupted in the extracellular space, resulting in Frag^C^ which lacks this polyarginine stretch, while this polyarginine stretch was reported to be a cell-penetrating signal sequence that enabled secreted PTENα to enter other cells [6]. Thus, our results indicate that Frag^C^ exerted its tumor-suppressive role in the extracellular space without entering the cells, although the underlying mechanisms remain to be explored. Of note, because there is no difference in the tumor-suppressive effects between wild-type PTENα (which is cleaved to generate both Frag^N^ and Frag^C^) and Frag^C^, we postulate that Frag^N^ did not play a role in tumorigenesis here.

Considering that genomic alterations such as mutation, deletion or amplification of the *PTEN* gene are not frequently observed in liver cancer [22], and liver cancer is one of the most prevalent life-threatening diseases in China [23], this cancer was chosen to study the function of PTENα/β. Previously, we proposed a tumor-promoting role of PTENα/β, which was dependent on its association with WDR5 in the nucleus [15]. Through the findings of this study, we propose a model that the overall role of PTENα in tumorigenesis is an additive effect of its intracellular tumor-promoting role and its extracellular tumor-suppressive role. The result of their wrestling decides whether PTENα manifests a tumor-promoting or tumor-suppressive role. We show that in liver cancer tissues, these two opposite roles of PTENα are differentially regulated in a way that favors tumor progression. While the intracellular tumor-promoting effect of PTENα is enhanced by the increased protein stability of PTENα resulted from the decreased expression of E3 ligase FBXW11 and increased expression of deubiquitinase USP9X as we previously reported [15], the extracellular tumor-suppressive role of PTENα was attenuated by the reduced generation of Frag^C^. Based on this, we reason that the role of PTENα in tumorigenesis might differ among different cellular contexts or cancer types, depending on the strength of its intracellular and extracellular roles and how they are regulated.

Proteolytic cleavage regulates numerous processes in health and disease. One key player is the ubiquitously expressed serine protease Furin, a prototypical and best-characterized member of the evolutionary ancient family of serine proteases known as the proprotein convertases subtilisin/kexin type (PCSK) [24]. Furin is localized in the trans-Golgi network and endosome compartments, at the cell surface and in the extracellular space [18, 20, 25, 26], which enables it to process a large variety of intra- and extracellular substrates including cytokines, hormones, growth factors and receptors in mammals [24], by cleaving them at polybasic recognition motifs [27]. We showed that Furin cleaved PTENα almost solely in the extracellular space, and the cleavage could not be detected in the cell. Visualization of intracellular PTENα and Furin revealed that the two are not in the same intracellular compartment, which might cause Furin to be unable to cleave intracellular PTENα.

Furin dysregulation, caused either by variations in Furin expression levels, enzymatic activity and/or cellular localization or even mutations in the cleavage site of a single Furin target protein, may have detrimental effects and is associated with a variety of disorders including cancer [20, 28–30]. However, the relative contribution of individual Furin substrate to tumor progression remains largely unclear. In agreement with its tumor-suppressive role, PTENα cleavage is substantially inhibited in liver cancer tissues compared to normal tissues, which might be partially attribute to the down-regulation of Furin in liver cancer tissues. Aberrant high expression or activation of Furin has been reported to promote the formation and progression of various malignancies including colon carcinoma, head and neck cancers, lung, skin and brain tumors [31]. However, we found that Furin was downregulated in liver cancer tissues compared to paired normal tissues and low *FURIN* expression predicted poor prognosis of liver cancer patients. In agreement with our findings, it has been reported that liver-specific inactivation of Furin leads to increased hepatocellular carcinoma growth [32], and Furin overexpression suppresses tumor growth and predicts a better postoperative disease-free survival in hepatocellular carcinoma [33].

By focusing on the NTE-dependent processing of extracellular PTENα/β, this study not only proposes a model that reconciles the paradoxical roles of extracellular and intracellular PTENα in tumorigenesis, but also provides more precise guidelines for therapeutic uses of PTENα as proposed previously [6], by revealing the tumor-suppressive role its C-terminal fragment in the extracellular space.

## Materials/Subjects and Methods

**Key resource table**

**Table:** 

**REAGENT or RESOURCE**	**SOURCE**	**IDENTIFIER**
**Antibodies**		
Rabbit anti-PTEN	CST	Cat#9559; RRID:AB_390810
Rabbit anti-β-actin	MBL	Cat# PM053-7; RRID:AB_10697035
Mouse anti-FLAG	Sigma-Aldrich	Cat# A8592; RRID:AB_439702
Rabbit anti-GFP	Abcam	Cat# ab183734; RRID:AB_2732027
Rabbit anti-Akt (pan)	CST	Cat#4691; RRID:AB_915783
Rabbit anti-Phospho-Akt (Ser473)	CST	Cat# 4060; RRID:AB_2315049
Rabbit anti-V5	Abcam	Cat# ab182008
Rabbit anti-WDR5	Abcam	Cat# ab178410
Mouse anti-S-tag	Beyotime	Cat# AF0285
Rabbit anti-Furin	Abcam	Cat# ab183495
**Recombant DNA**		
pLVX-IRES-ZsGREEN1-PTENα	This Paper	N/A
pLVX-IRES-ZsGREEN1-PTENβ	This Paper	N/A
pLVX-IRES-ZsGREEN1-PTEN	This Paper	N/A
pLVX-IRES-ZsGREEN1-PTENα-R49K	This Paper	N/A
pLVX-IRES-ZsGREEN1-PTENα-R49A	This Paper	N/A
pLVX-IRES-ZsGREEN1-PTENα-3RK	This Paper	N/A
pLVX-IRES-ZsGREEN1-EGFP	This Paper	N/A
pLVX-IRES-ZsGREEN1-N146-EGFP	This Paper	N/A
pLVX-IRES-ZsGREEN1-N146-EGFP(Δ39–40)	This Paper	N/A
pLVX-IRES-ZsGREEN1-N146-EGFP(Δ65–72)	This Paper	N/A
pLVX-IRES-ZsGREEN1-N146-EGFP(Δ75–84)	This Paper	N/A
pLVX-IRES-ZsGREEN1-N146-EGFP(Δ98–108)	This Paper	N/A
pLVX-IRES-ZsGREEN1-N146-EGFP(Δ112–123)	This Paper	N/A
pLVX-IRES-ZsGREEN1-N146-EGFP(Δ126–141)	This Paper	N/A
pQCXIN-3×Flag-PTENα	This Paper	N/A
pQCXIN-PTENα-3×Flag	This Paper	N/A
pQCXIN-PTENα-3×Flag (Δ41–50)	This Paper	N/A
pQCXIN-PTENα-3×Flag (Δ51–60)	This Paper	N/A
pQCXIN-PTENα-3×Flag (Δ61–70)	This Paper	N/A
pQCXIN-PTENα-3×Flag (Δ71–80)	This Paper	N/A
pQCXIN-PTENα-3×Flag (Δ81–90)	This Paper	N/A
pQCXIN-PTENα-3×Flag (Δ6R)	This Paper	N/A
pQCXIN-PTENα-R47,49K-3×Flag	This Paper	N/A
pQCXIN-PTENα-R50,51K-3×Flag	This Paper	N/A
pQCXIN-PTENα-R47A-3×Flag	This Paper	N/A
pQCXIN-PTENα-R49A-3×Flag	This Paper	N/A
pQCXIN-3×Flag-PTENα-3×Flag	This Paper	N/A
pQCXIN-3×Flag-PTENα-3×Flag (Δ6R)	This Paper	N/A
pQCXIN-3×Flag-PTENα-3×Flag (Δ6A)	This Paper	N/A
pQCXIN-PTENα-3×Flag (Δ66–67)	This Paper	N/A
pQCXIN-PTENα-3×Flag (Δ92–99)	This Paper	N/A
pQCXIN-PTENα-3×Flag (Δ102–111)	This Paper	N/A
pQCXIN-PTENα-3×Flag (Δ125–135)	This Paper	N/A
pQCXIN-PTENα-3×Flag (Δ139–150)	This Paper	N/A
pQCXIN-PTENα-3×Flag (Δ153–168)	This Paper	N/A
pQCXIN-3×Flag-PTENβ	This Paper	N/A
pQCXIN-3×Flag-PTENβ-Δ6R	This Paper	N/A
pQCXIN-3×Flag-PTENβ-3×Flag	This Paper	N/A
pET-32a-TrxA-S-tag-PTEN	This Paper	N/A
pET-32a-TrxA-S-tag-PTENα	This Paper	N/A
pET-32a-TrxA-S-tag-PTENα−Δ6R	This Paper	N/A
pET-32a-TrxA-S-tag-PTENα−Frag^C^	This Paper	N/A
pET-32a-TrxA-S-tag-Flag	This Paper	N/A
pET-32a-TrxA-S-tag-PTENα-Flag	This Paper	N/A
pLVX-Puro-Furin-Flag	This Paper	N/A
pLX304-Furin-V5	This Paper	N/A
pEGFP-N1-PTENα	This Paper	N/A
pEGFP-N1-PTENαR49A	This Paper	N/A
pEGFP-N1-PTENαR49K	This Paper	N/A
pEGFP-N1-PTENα3RK	This Paper	N/A
pEGFP-N1-PTENα−Δ6R	This Paper	N/A
**Chemicals, peptides, and recombinant proteins**		
TrxA-S-tag-PTEN	This Paper	N/A
TrxA-S-tag-PTENα	This Paper	N/A
TrxA-S-tag-Flag	This Paper	N/A
TrxA-S-tag-PTENα-Flag	This Paper	N/A
PTENα	This Paper	N/A
PTENαΔ6R	This Paper	N/A
PTENα-Frag^C^	This Paper	N/A
**REAGENT or RESOURCE**	**SOURCE**	**IDENTIFIER**
HSA	Sino Biological	Cat# 10968-HNAY
T4 DNA Ligase	New England Biolabs	M0202T
TRIzol™ Reagent	Thermo	Cat# 15596026
MMLV reverse transcription reagent	Promega	Cat# M1705
NEBuilder HiFi DNA Assembly Master Mix	New England Biolabs	E2621L
**Oligonucleotides**		
sh*FURIN* targeting sequence: GGCCTTCATGACAACTCATTC	This Paper	N/A
*FURIN* (F)-QPCR primer GAGCCCAAAGACATCGGGAA	This Paper	N/A
*FURIN* (R)-QPCR primer GCCACGGCGATTATAGGACA	This Paper	N/A
*GAPDH* (F)-QPCR primer GAAGGTGAAGGTCGGAGTCAA	This Paper	N/A
*GAPDH* (R)-QPCR primer GCTCCTGGAAGATGGTGATG	This Paper	N/A
sgPTEN targeting sequence: AAACAAAAGGAGATATCAAG	This Paper	N/A
**Experimental models: cell lines**		
Homo sapiens: 293 T	Cell Bank of Chinese Academy of Sciences	GNHu17
Homo sapiens: SMMC-7721	Cell Bank of Chinese Academy of Sciences	TCHu 52
Homo sapiens: SW620	Cell Bank of Chinese Academy of Sciences	TCHu101
Homo sapiens: HuH-7	Cell Bank of Chinese Academy of Sciences	TCHu182
Homo sapiens: MiaPaCa2	Cell Bank of Chinese Academy of Sciences	SCSP-568
Homo sapiens: H441	ATCC	HTB-174
**Software and Algorithms**		
GraphPad Prism, Version 7.0.0 for windows	GraphPad	https://www.graphpad.com
Image J	NIH, USA	https://imagej.nih.gov/ij

### Human cell lines

293 T, SMMC-7721, HuH-7, and MiaPaCa2 cells were maintained in Dulbecco’s Modified Eagle’s Medium (DMEM) supplemented with 10% fetal bovine serum (FBS). SW620 and NCI-H441 were maintained in RPMI-1640 supplemented with 10% FBS. No signs of mycoplasma contamination were found for all cell lines. Short tandem repeat profiling was used for cell line authentication.

### Mouse studies

A total of 1×10^6^ cells in 100 μl serum-free media were implanted subcutaneously into 4-6 weeks old female nude mice (Shanghai Laboratory of Animal Center, Chinese Academy of Sciences) and tumor volume was assessed by calipers. Volumetric measurements were made using length × (width)^2^/2. According to animal care and enforcement, the largest subcutaneous tumor mass on one flank was less than 1 cm^3^. Animal care and experiments were in agreement with all of the animal research-related ethical regulations under the approvement of the committee for humane treatment of animals at Shanghai Jiao Tong University School of Medicine.

### Human samples

Human samples were obtained with informed consent from human tissue bank of Renji Hospital of Shanghai Jiao Tong University School of Medicine (SJTU-SM) under approval of the Medical Ethic Committee of SJTU-SM. Information on samples of liver cancer patients are shown in Supplemental Spreadsheet 1.

### Plasmids construction

The PTEN, PTENα and PTENβ were PCR amplified from cDNA and inserted into pLVX-IRES-ZsGREEN1, pQCXIN or pEGFP-N1 expression vectors. Mutagenesis or truncations were formed by PCR. PTEN and PTENα were inserted into pET-32a vector for protein purification. The Furin plasmids were purchased from Core Facility of Basic Medical Sciences, Shanghai Jiao Tong University School of Medicine. The oligonucleotides for shRNA targeting *FURIN* were cloned into pLKO.1-Puro vectors. The empty pLKO.1-Puro vector was used as a negative control.

### Immunoprecipitation

The cells were collected and washed in cold 1 × PBS and lysed using RIPA (Millipore) with 1 mM PMSF, and 1 × protease inhibitor cocktail (Calbiochem) as per manufacturer’s protocol after transfection. The whole-cell lysates were incubated for 4 h at 4 °C with anti-Flag M2 Affinity Gel or GFPTrap (ChromoTek) in ratio recommended by manufacturer for immunoprecipitation.

### Western blot

For detection of most proteins, protein extracts were separated by SurePAGE, Bis-Tris, 4–12% gel (GenScript). Specifically, for the detection of endogenous Frag^C^, 10% SDS-PAGE was used. After separation, proteins were transferred to nitrocellulose membrane (Bio-Rad, Richmond, CA), blocked by 5% nonfat milk for 1 h at room temperature and sequentially incubated in primary antibody in 2% BSA overnight at 4 °C. The following day, blots were washed in TBST and incubated in horseradish peroxidase (HRP)-linked secondary antibody (Cell Signaling, Beverly, MA) in 2% BSA for 1 h at room temperature. Immobilon Western Chemiluminescent HRP substrate kit (Merck Millipore) was used for detection. Full and uncropped Western blots are provided in Original Data File.

### Transfection and Secretion determination

Transfection of DNA constructs into 293 T and SMMC-7721 cells was performed using jetPRIME (Polyplus) according to the manufacturer’s protocols. For determination of protein secretion, cells were replaced with DMEM for 16 hours. The medium was filtered through a 0.22 μm filter before being concentrated by a 3 kD Amicon filter (Millipore) and cell lysate was collected.

### Protein expression and Purification

Recombinant PTEN, PTENα or its derivatives were expressed in *Escherichia coli* Rosetta2 (DE3) (TSINGKE) using the pET-32a vector expression system. After induction for 18 hours with 0.1 mM IPTG at 18 °C, the cells were harvested by centrifugation and the pellets were resuspended in lysis buffer (25 mM Tris-HCl, pH 7.5, 500 mM NaCl, 10% glycerol, 1 mM PMSF, 5 mM benzamidine, 1 μg/mL leupeptin and 1 μg/mL pepstatin). The cells were then lysed by sonication and the cell debris was removed by ultracentrifugation. The supernatant was mixed with BeyoGold™ His-tag Purification Resin (Beyotime) and rocked for 2 hours at 4 °C before elution with 300 mM imidazole. The N terminus tag of the proteins intratumorally injected were removed through protease 3 C digestion. The proteins were further purified by gel-filtration chromatography equilibrated with 25 mM Tris-HCl, pH 7.5, 150 mM NaCl, 1 mM DTT. The purified proteins were concentrated by centrifugal filtrations (Millipore), then stored in aliquots at −80 °C.

### In vitro cleavage of PTENα with Furin

The recombinant forms of PTEN and PTENα with N-terminal TrxA-S-tag were expressed in *Escherichia coli* Rosetta2 (DE3) (TSINGKE) and purified using BeyoGold™ His-tag Purification Resin (Beyotime). Furin-Flag was purified with Anti-Flag M2 Affinity Gel from 293 T cells. For cleavage assay, purified Furin-Flag was incubated with PTEN or PTENα separately in cleavage buffer (25 mM Tris-HCl, 150 mM NaCl, 2.5 mM CaCl_2_, pH 7.4) for 1 h at 37 °C.

### Mass spectrometry

For in vitro PTENα cleavage site identification experiments, the purified Furin-Flag was incubated with bacterially purified PTENα in cleavage buffer for 1 h at 37 °C, then the proteins were loaded on SurePAGE, Bis-Tris, 4–12% gel (GenScript). After Coomassie blue staining, gel slices with a molecular weight corresponding to Frag^N^ were digested in-gel by chymotrypsin (Promega) overnight at 25 °C. The extracted peptides were desalted by ziptip C18 and dried by vacuum centrifugation, and dissolved to 10 μl of 2% ACN and 0.1% formic acid. The peptides identification was performed on an Orbitrap Fusion LUMOS mass spectrometer (Thermo Fisher Scientific) connected to an Easy-nLC 1200 via an Easy Spray (Thermo Fisher Scientific). The peptides were loaded onto a self-packed analytical PicoFrit column with integrated spray tip (New Objective, Woburn, MA, USA) (75 μm × 15 cm length) packed with ReproSil-Pur 120 A C18-AQ 1.9 μm (Dr. Maisch GmbH, Ammerbuch, Germany) and separated within a 60 minutes linear gradient from 95% solvent A (0.1% formic acid/2% acetonitrile/98% water) to 28% solvent B (0.1% formic acid/80% acetonitrile) at a flow rate of 300 nl/min at 50 °C. The mass spectrometer was operated in positive ion mode and employed in the data-dependent mode within the specialized cycle time (2 S) to automatically switch between MS and MS/MS. One full MS scan from 350 to 1500 m/z was acquired at high resolution R = 120,000 (defined at m/z = 400); MS/MS scans were performed at a resolution of 30,000 with an isolation window of 1.6 Da and higher energy collisional dissociation (HCD) fragmentation with collision energy of 30% +/− 5. Dynamic exclusion was set to 30 s. All MS/MS ion spectra were analyzed using PEAKS 10.6 (Bioinformatics Solutions) for processing, de novo sequencing and database searching. Resulting sequences were searched against the PTENα sequence with mass error tolerances of 10 ppm and 0.02 Da for parent and fragment, respectively, the digest mode specified as Chymotrypsin digestion, and Carbamidomethylation of Cysteine (C + 57.02) specified as fixed modifications and Oxidation of methionine (M + 15.99) as variable modification. FDR estimation (<1%) was enabled. Peptides were filtered for −10 log *P* ≥ 20 (*P* < 0.01).

### CRISPR-Cas9

For the generation of *PTEN*-knockout cell lines in 293 T or SMMC-7721, the cells were transfected with LentiCRISPR v2 containing *PTEN* targeting sequence (sgRNA targeting sequence: ACAAAAGGAGATATCAAGAGG). Puromycin was used to select positive cells. Then the cells were diluted and single colonies were isolated. The effect of gRNA was detected by Western blot. PCR and sequencing were used to confirm homozygous editing of the gene loci.

### Human liver cancer samples

Human samples were used for Western blot analysis. Quantification of protein bands was performed using ImageJ software. The diagnosis of normal tissue or liver cancer was confirmed by independent pathologists based on histological findings. All experiments were performed with informed consent obtained from all subjects with the approval of the Medical Ethic Committee of Shanghai Jiao Tong University School of Medicine Review Board.

### qRT-PCR

Total RNA from indicated cells was extracted with Trizol (Invitrogen) according to the manufacturer’s protocol. RNA was digested with DNase I (Promega), reverse transcribed to cDNA using random primers (Takara) and M-MLV Reverse Transcriptase (Promega, Fitchburg, WI), followed by qRT-PCR with the SYBR Green PCR Master Mix (Applied Biosystems, Foster City, CA). Expression levels of each gene were normalized to *GAPDH* and calculated relative to the control.

### Protein labeling and delivery

The recombinant forms of PTEN and PTENα derivatives were labeled by Alexa Fluor 647 protein labeling system (Invitrogen) according to manufactory’s instruction. The fluorescein-conjugated protein was complexed with PULSin for 15 min and added to *PTEN*-knockout SMMC-7721 cells. Cells are observed by Nikon Eclipse TI Laser Scanning Microscope after 20 h.

### Tumor treatment

Once tumors reached 50–100 mm^3^, the mice were randomized into four groups. PTENα or its derivatives or HSA (10 μg/100 μL) were intratumorally injected once/day for 6 days. Tumor volume was assessed by calipers.

### Quantification and statistical analysis

The ways of quantification of each experiment have been provided in the Method Details. The statistical information of each experiment, including the statistical methods, the *P* values and numbers (n), were shown in the figures and corresponding legends.

### Reporting summary

Further information on research design is available in the Nature Research Reporting Summary linked to this article.

## Supplementary information

Original Data

Supplementary Figures

Reporting Summary

Supplemental Spreadsheet 1

## Data Availability

All data are available in this manuscript and supplementary files. Further information and requests for resources and reagents should be directed to and will be fulfilled by Shao-Ming Shen (smshen@shsmu.edu.cn).
