# Supplementary material for: Furin extracellularly cleaves secreted PTENα/β to generate C-terminal fragment with a tumor-suppressive role
Source: Cell Death Dis. 2022 Jun 6;13(6):532. doi: 10.1038/s41419-022-04988-2 (PMC9170693; doi:10.1038/s41419-022-04988-2)
Supplement: Supplementary file 1 — Original Data [file 41419_2022_4988_MOESM1_ESM.pptx]

## Slide 1
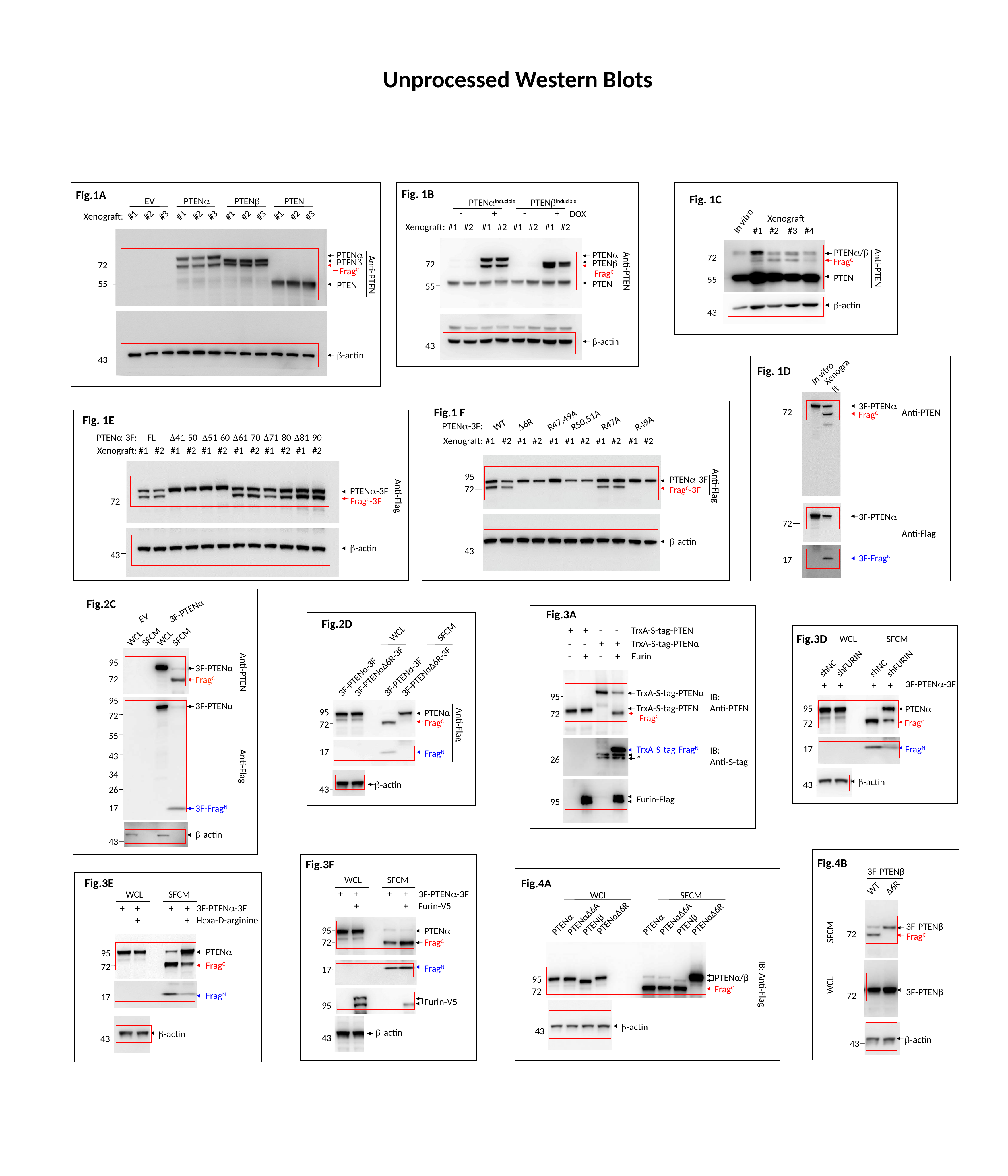

Unprocessed Western Blots
Fig.1A
EV
PTENα
PTENβ
PTEN
#2
#2
#2
#2
#1
#1
#1
#1
#3
#3
#3
#3
Xenograft:
PTENα
PTENβ
72
FragC
Anti-PTEN
55
PTEN
β-actin
43
Fig. 1B
PTENαinducible
PTENβinducible
−
+
−
+
DOX
Xenograft:
#1
#2
#1
#2
#1
#2
#1
#2
PTENα
PTENβ
72
Anti-PTEN
FragC
PTEN
55
β-actin
43
Fig. 1C
Xenograft
In vitro
#1
#2
#3
#4
PTENα/β
72
FragC
Anti-PTEN
PTEN
55
β-actin
43
Xenograft
In vitro
Fig. 1D
3F-PTENα
72
Anti-PTEN
FragC
3F-PTENα
72
Anti-Flag
3F-FragN
17
Fig.1 F
R47,49A
R50,51A
R49A
R47A
Δ6R
WT
PTENα-3F:
Xenograft:
#1
#2
#1
#2
#1
#1
#2
#1
#2
#1
#2
95
PTENα-3F
Anti-Flag
72
FragC-3F
β-actin
43
Fig. 1E
PTENα-3F:
FL
Δ41-50
Δ51-60
Δ61-70
Δ71-80
Δ81-90
Xenograft:
#1
#2
#1
#2
#1
#2
#1
#2
#1
#2
#1
#2
PTENα-3F
Anti-Flag
FragC-3F
72
β-actin
43
Fig.2C
3F-PTENα
EV
SFCM
SFCM
WCL
WCL
95
3F-PTENα
Anti-PTEN
FragC
72
95
3F-PTENα
72
55
43
Anti-Flag
34
26
3F-FragN
17
β-actin
43
Fig.3A
+
+
-
-
TrxA-S-tag-PTEN
-
-
+
+
TrxA-S-tag-PTENα
-
+
-
+
Furin
TrxA-S-tag-PTENα
95
IB:
Anti-PTEN
TrxA-S-tag-PTEN
72
FragC
IB:
Anti-S-tag
TrxA-S-tag-FragN
*
26
Furin-Flag
95
Fig.2D
SFCM
WCL
3F-PTENαΔ6R-3F
3F-PTENαΔ6R-3F
3F-PTENα-3F
3F-PTENα-3F
PTENα
95
FragC
72
Anti-Flag
17
FragN
β-actin
43
Fig.3D
WCL
SFCM
shFURIN
shFURIN
shNC
shNC
3F-PTENα-3F
+
+
+
+
95
PTENα
FragC
72
FragN
17
β-actin
43
Fig.4B
3F-PTENβ
Δ6R
WT
3F-PTENβ
SFCM
72
FragC
WCL
3F-PTENβ
72
β-actin
43
Fig.3F
WCL
SFCM
+
+
+
+
3F-PTENα-3F
+
+
Furin-V5
95
PTENα
FragC
72
FragN
17
Furin-V5
95
β-actin
43
Fig.4A
WCL
SFCM
PTENαΔ6A
PTENαΔ6R
PTENαΔ6A
PTENαΔ6R
PTENα
PTENα
PTENβ
PTENβ
PTENα/β
95
IB: Anti-Flag
FragC
72
β-actin
43
Fig.3E
WCL
SFCM
+
+
+
+
3F-PTENα-3F
+
+
Hexa-D-arginine
PTENα
95
FragC
72
FragN
17
β-actin
43

## Slide 2
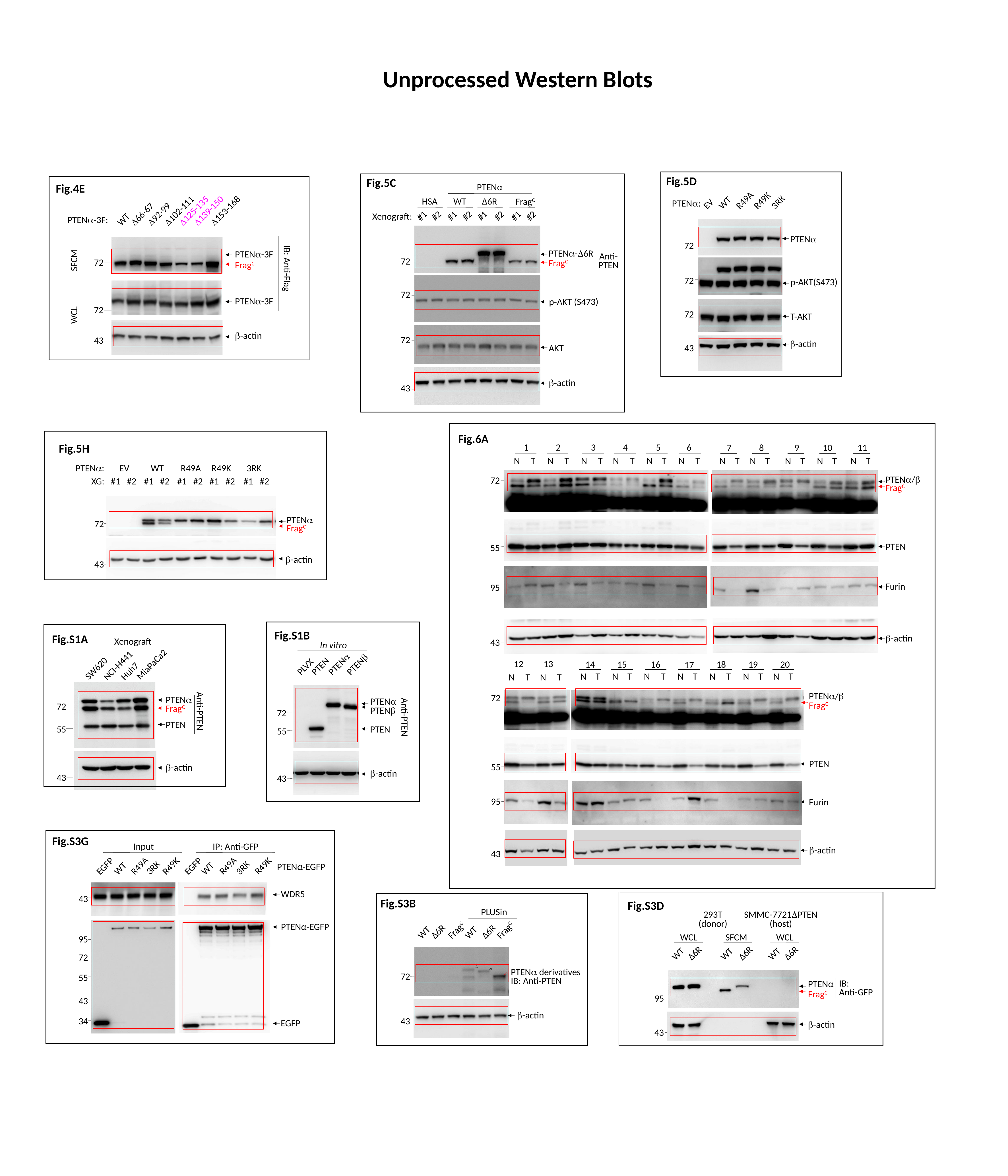

Unprocessed Western Blots
Fig.5D
R49A
R49K
3RK
WT
PTENα:
EV
PTENα
72
72
p-AKT(S473)
72
T-AKT
β-actin
43
Fig.5C
PTENα
HSA
WT
Δ6R
FragC
#2
#2
#2
#1
#1
#1
#2
#1
Xenograft:
PTENα-Δ6R
Anti-PTEN
72
FragC
72
p-AKT (S473)
72
AKT
β-actin
43
Fig.4E
Δ139-150
Δ153-168
Δ102-111
Δ125-135
WT
Δ66-67
Δ92-99
PTENα-3F:
PTENα-3F
SFCM
72
FragC
IB: Anti-Flag
PTENα-3F
72
WCL
β-actin
43
Fig.6A
Fig.5H
PTENα:
EV
WT
R49A
R49K
3RK
XG:
#1
#2
#1
#2
#1
#2
#1
#2
#1
#2
PTENα
72
FragC
β-actin
43
1
2
3
4
5
6
7
8
9
10
11
N
T
N
T
N
T
N
T
N
T
N
T
N
T
N
T
N
T
N
T
N
T
PTENα/β
FragC
PTEN
Furin
β-actin
12
13
14
15
16
18
19
20
17
N
T
N
T
N
T
N
T
N
T
N
T
N
T
N
T
N
T
PTENα/β
FragC
PTEN
Furin
β-actin
72
55
95
Fig.S1B
In vitro
PTENβ
PTENα
PTEN
PLVX
PTENα
PTENβ
72
Anti-PTEN
PTEN
55
β-actin
43
Fig.S1A
Xenograft
MiaPaCa2
NCI-H441
SW620
Huh7
PTENα
72
FragC
Anti-PTEN
PTEN
55
β-actin
43
43
72
55
95
Fig.S3G
Input
IP: Anti-GFP
R49A
R49K
3RK
WT
EGFP
EGFP
R49K
R49A
PTENα-EGFP
3RK
WT
WDR5
43
PTENα-EGFP
95
72
55
43
34
EGFP
43
Fig.S3D
293T (donor)
SMMC-7721ΔPTEN (host)
WCL
WCL
SFCM
Δ6R
WT
Δ6R
Δ6R
WT
WT
PTENα
IB:
Anti-GFP
FragC
95
β-actin
43
Fig.S3B
PLUSin
FragC
FragC
Δ6R
WT
WT
Δ6R
PTENα derivatives
IB: Anti-PTEN
72
β-actin
43
