# Supplementary material for: Furin extracellularly cleaves secreted PTENα/β to generate C-terminal fragment with a tumor-suppressive role
Source: Cell Death Dis. 2022 Jun 6;13(6):532. doi: 10.1038/s41419-022-04988-2 (PMC9170693; doi:10.1038/s41419-022-04988-2)
Supplement: Supplementary file 2 — Supplementary Figures [file 41419_2022_4988_MOESM2_ESM.docx]

**Supplementary Figures**

**
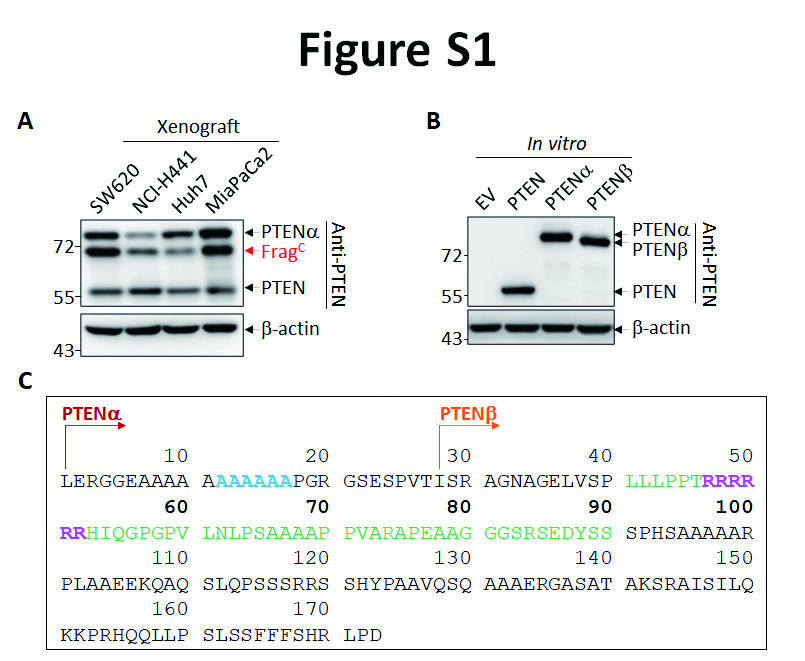
**

**Figure S1. Related to Figure 1**

(A) Western blot analysis for the indicated proteins in xenografts derived from SW620, NCI-H441, Huh7, and MiaPaCa2 cells stably expressing PTENα.

(B) Western blot analysis for the indicated proteins in the cell lysates of *in vitro* cultured *PTEN*-knockout SMMC-7721 cells stably expressing PTEN, PTENα or PTENβ.

(C) Amino acid sequence of the 173-residue NTE of PTENα with the translational initiation codons of PTENα and PTENβ respectively indicated.


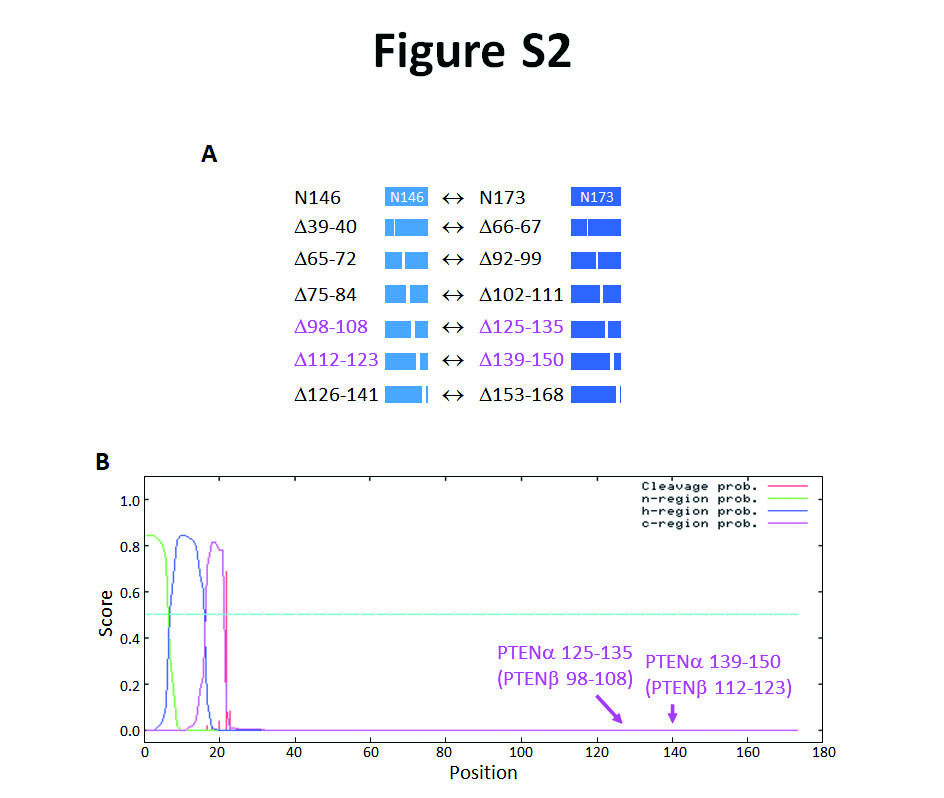


**Figure S2. Related to Figure 4**

(A) Schematics of deletions made to N146 and their corresponding locations in N173.

(B) SignalP (http://www.cbs.dtu.dk/services/SignalP-3.0) prediction of signal peptide sequence in N173.


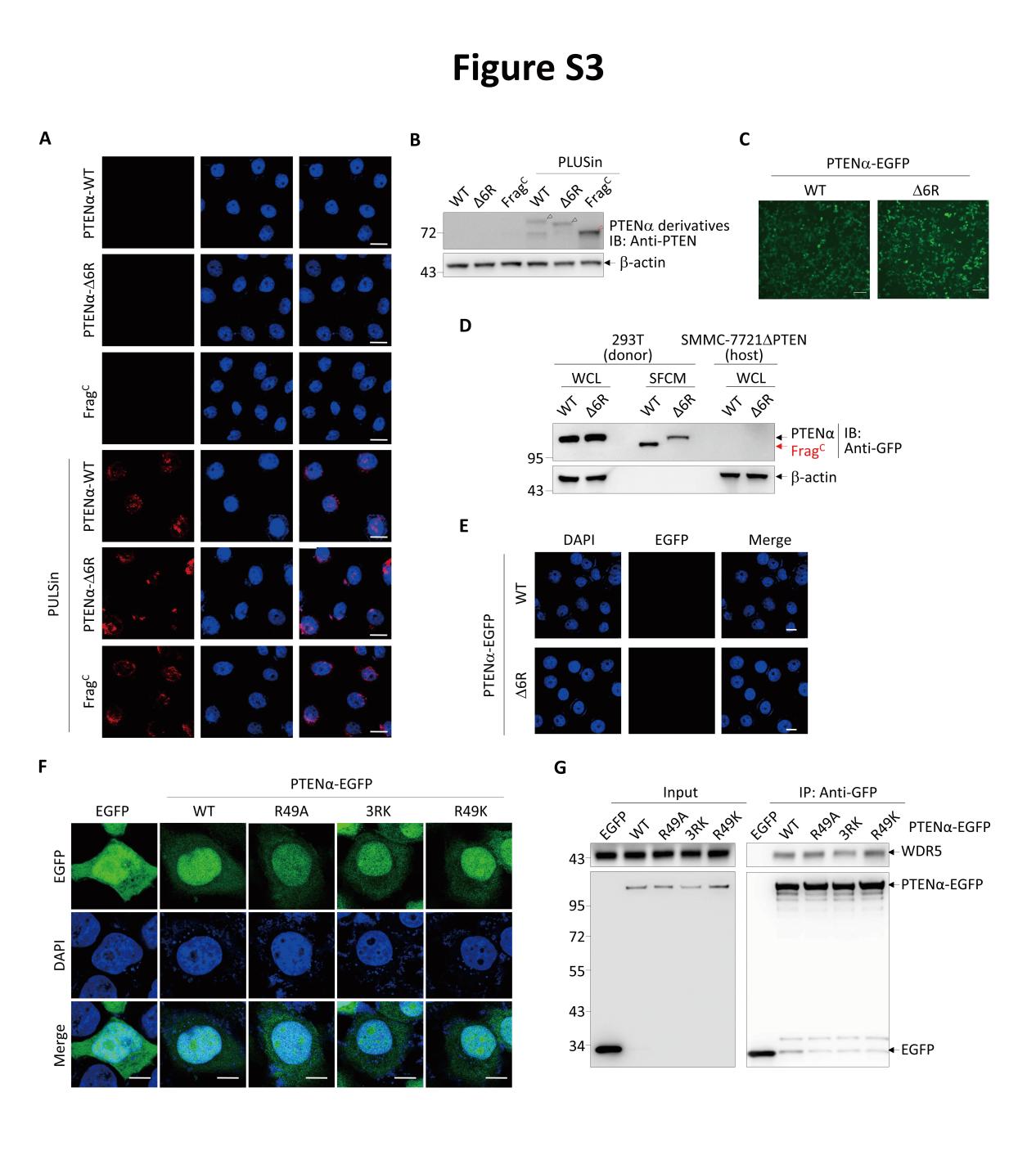


**Figure S3. Related to Figure 5.**

(A) *PTEN*-knockout SMMC-7721 cells were treated by fluorescein-conjugated PTENα derivatives in the absence or presence of PULSin, and representative images of fluorescein-conjugated proteins in *PTEN*-knockout SMMC-7721 cells with re-staining of DAPI were shown. Scale bar represents 20 μm.

(B) Western blot analysis of indicated proteins in *PTEN*-knockout SMMC-7721 cells treated by fluorescein-conjugated PTENα derivatives in the absence or presence of PULSin.

(C-E) 293T cells were transfected with EGFP-tagged PTENα-WT or PTENαΔ6R and the culture medium were collected and used to treat *PTEN*-knockout SMMC-7721 cells for 12 hours. Representative images of EGFP-tagged proteins in 293T cells with scale bar representing 100 μm (C). Western blot analysis for the indicated proteins in the WCL and SFCM of 293T cells and in the WCL of culture medium-treated *PTEN*-knockout SMMC-7721 cells (D). Representative images of EGFP-tagged proteins in culture medium-treated *PTEN*-knockout SMMC-7721 cells with re-staining of DAPI with scale bar representing 20 μm (E).

(F) Representative images of EGFP-tagged PTENα derivatives transfected in *PTEN*-knockout SMMC-7721 cells with re-staining of DAPI. Scale bar represents 10 μm.

(G) Western blot analysis of indicated proteins in the immunoprecipitates of GFP-tagged proteins transfected in *PTEN*-knockout SMMC-7721 cells.
